# Mitochondria-Associated Membranes in Aging and Senescence

**DOI:** 10.14336/AD.2024.0652

**Published:** 2025-08-05

**Authors:** Zhaojia Wang, Xiao Du, Shiran Yu, Xuzhen Yan, Yanguo Xin

**Affiliations:** ^1^Department of Cardiology, Beijing Friendship Hospital, Capital Medical University, Beijing, China.; ^2^Experimental and Translational Research Center, Beijing Friendship Hospital, Capital Medical University, Beijing, China.

**Keywords:** Aging, mitochondria, ER, contact site, MAMs

## Abstract

Although the pursuit of eternal youth remains elusive, progress in the fields of medicine and science has greatly extended the human lifespan. Nevertheless, the rising incidence of diseases and their economic impact present notable obstacles. Mitochondria-associated membranes (MAMs), essential sites for close interaction between mitochondria and the endoplasmic reticulum (ER), are increasingly recognized for their involvement in both normal cellular processes and the development of diseases. Studies suggest that MAMs undergo dynamic alterations, particularly pertinent in the investigation of age-related illnesses. This review highlights the significance of MAMs in age-related conditions, elucidating the morphological and functional alterations in mitochondria and ER during aging. By emphasizing the complex interaction between these organelles, it demonstrates the cell's adaptive responses to combat age-related deterioration. Suggesting MAMs as potential targets for therapeutic interventions holds the potential for attenuating the progression of age-related diseases.

## Introduction

Significant advancements in medical and scientific research have led to a notable increase in human life expectancy. Despite the benefits of longer life spans, challenges arise, such as the decreased ability to activate defense mechanisms that mitigate cellular stress. Combined with environmental exposures, this results in a diminished capacity to repair cellular damage [1, 2]. As a result, aging becomes a primary risk factor for the development and progression of various pathological conditions. Understanding the underlying mechanisms of aging becomes imperative in addressing these issues.

Aging is a complex process marked by the progressive decline in cellular, tissue, and organismal functions. At the cellular level, aging is closely associated with oxidative stress, Deoxyribo Nucleic Acid (DNA) modifications, proteostasis impairment, and organelle turnover inefficiency [3, 4]. Aging can be understood as the progressive imbalance between the accumulation of stochastic damage and the resilience mechanisms that continuously repair such damage, ultimately leading to the onset of chronic diseases, frailty, and mortality. The primary objectives are to elucidate the mechanisms and pathways involved in the aging process and to offer a comprehensive view of potential therapeutic targets. These targets would pave the way for developing therapeutic strategies to mitigate the progression of age-related diseases.

The progressive decline in interrelated cellular functions, resulting in heightened organismal damage, is a fundamental aspect of the aging process. Research indicates that changes in intracellular organelle function are crucial in governing diverse biological processes during aging. The intimate association between organelles enables communication through specialized connections called membrane contact sites between neighboring organelles. Significantly, MAMs play a crucial role in integrating and coordinating various cellular processes such as oxidative stress, mitochondrial dynamics, and calcium (Ca^2+^) signaling, all of which demonstrate early age-related alterations [5, 6]. Dysfunction of MAMs has been associated with a range of pathological conditions, including neurodegenerative diseases, obesity, and cardiovascular disorders, among others.

## Discovery of ER-mitochondria contact sites and MAMs

**Figure 1.**
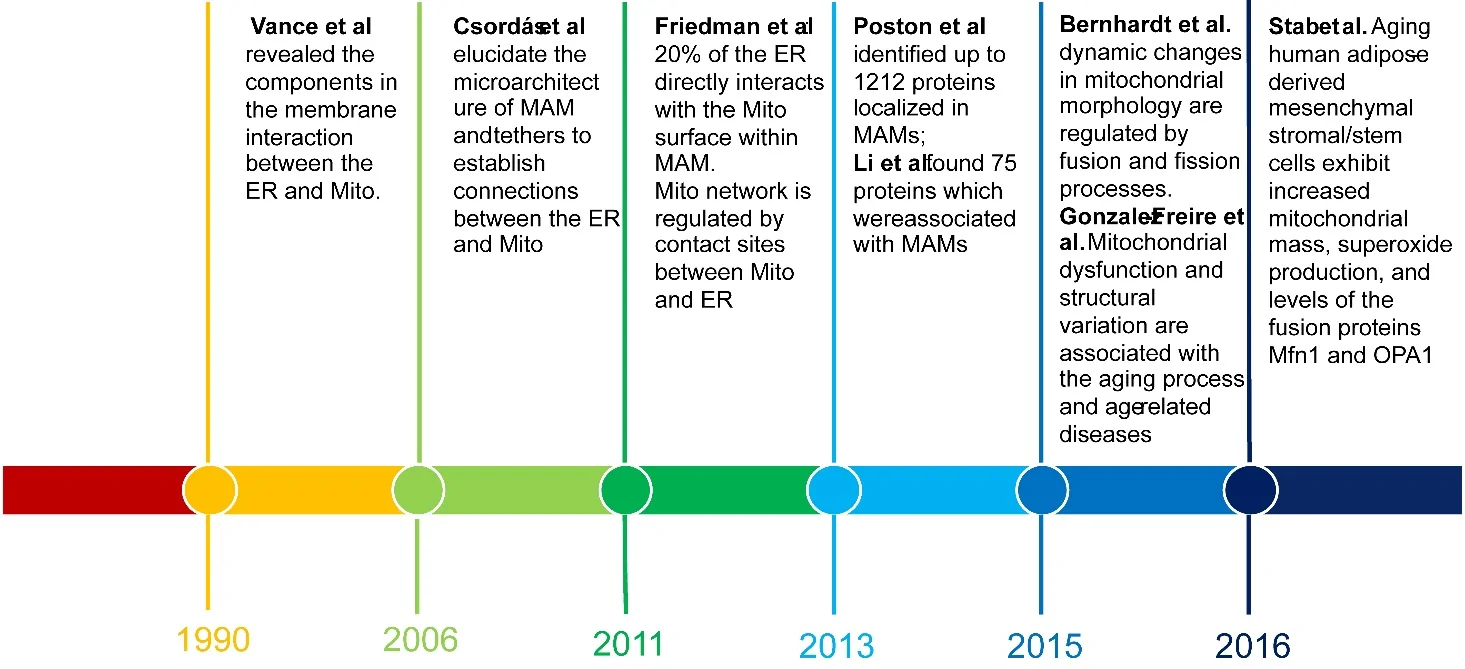
The historical timeline of the most important experimental observations and key discoveries in the course of studies of MAMs.

In 1990, Vance et al. [7] provided a detailed analysis of the components involved in the membrane interaction between the ER and mitochondria in rat liver. Following this, researchers have utilized a combination of electron microscopy and cell fluorescence microscopy methodologies to elucidate the microarchitecture of MAMs (Fig. 1). Utilizing electron tomography, it was revealed that tethers establish connections between the ER and mitochondria, with a distance of approximately 25 nm for rough ER and approximately 10 nm for smooth ER [8]. Wide-field digital 3D deconvolution microscopy observations have demonstrated that approximately 20% of the ER directly interacts with the mitochondrial surface within MAMs [9]. The frequency and spatial arrangement of MAMs are subject to variation due to the dynamic nature of these organelle interactions, which can exhibit significant variability under different cellular physiological conditions. The dynamic and adaptable properties of MAMs contribute to their highly variable composition. A proteomic analysis of MAMs components identified up to 1212 proteins localized in MAMs [10]. Following the exclusion of contaminated samples, approximately 75 proteins, including sorting proteins, chaperones, and protein kinases, were found to be associated with MAMs [11, 12].

Mitochondrial dysfunction and structural variations have been associated with the aging process and age-related disorders [13, 14]. The dynamic changes in mitochondrial morphology, transitioning from fragmented to filamentous networks, are regulated by fusion and fission processes essential for maintaining mitochondrial quality control [15]. Elevated levels of mitochondrial fusion proteins Mitofusin 1/2 (*Mfn1* and *Mfn2)* have been detected in aging skeletal muscle, indicating a potential increase in fusion activity in response to the accumulation of mutations in mitochondrial DNA [16, 17]. The observed elevation in mitochondrial fusion is concurrent with a decrease in the expression of the fission protein mitochondrial fission protein 1 (*Fis1)*. Notably, the restructuring of the mitochondrial network is regulated by contact sites between the mitochondria and the ER, which serve as loci for mitochondrial fission [9]. Furthermore, senescent human adipose-derived mesenchymal stromal/stem cells exhibit increased levels of mitochondrial mass, superoxide production, and fusion proteins *Mfn1* and optic atrophy 1 (*OPA1)* in comparison to younger cells at earlier passages [18]. These findings suggest that alterations in mitochondrial morphology observed in aging cells may be attributed to dysregulated processes of fission and fusion (Fig. 4).

## Misfolded protein aggregates present in MAMs

The dysregulation of proteostasis, characterized by a decline in protein degradation within cells, is a prominent feature of the aging process. This results in the accumulation of aggregates comprised of damaged or misfolded proteins, ultimately leading to cellular degeneration and the development of various pathologies. Recent research indicates that mitochondria may be involved in the uneven distribution of harmful protein aggregates during cell division in yeast [19-21], providing a potential mechanism for rejuvenation of the offspring. Specifically, older mother cells retain cellular waste, while younger buds can maintain a relatively clean cellular environment. The aggregates have been observed to interact with the surface of the ER and accumulate at MAMs, suggesting a potential involvement of MAMs in the regulation of protein quality control [19]. A comparable occurrence has been noted in immortalized human mammary epithelial stem-like cells undergoing asymmetric division, in which recently synthesized mitochondria exhibit a preference for segregation to daughter cells that retain stemness properties, whereas daughter cells inheriting older mitochondria differentiate into specialized cells. Subsequent studies employing the split-green fluorescent protein (GFP) system in human retinal pigment epithelial-1 (RPE1) cells and yeast have demonstrated that cytosolic proteins susceptible to aggregation are translocated into mitochondria for breakdown by mitochondrial proteases, including proto-oncogene, serine/threonine kinase (*Pim1)* [20]. These results indicate that mitochondria play a role in both the sequestration and degradation of protein aggregates.

In senescent cells, there is an observed increase in the unfolded protein response in the endoplasmic reticulum (UPR^ER^) among fibroblasts. Specifically, Druelle et al. [22] demonstrated that the activation of the transcription factor-6 (*ATF6*) plays a crucial role in determining the alterations seen in the senescent phenotype of Normal Human Fibroblasts (NHF) [23]. Additionally, this research group identified that *ATF6* regulates the intracrine cyclooxygenase-2/prostaglandin E2 pathway in two types of senescent cells. This suggests that while UPR^ER^ can impact markers of cellular senescence, its effects may vary depending on the specific effectors involved [24]. To enhance our understanding of the role of UPR^ER^ in cellular senescence, Pluquet et al. [25] provide a comprehensive review of the subject. During the aging process, organisms often accumulate intracellular aggregates comprised of unfolded proteins, which is associated with a notable reduction in the proteostasis network's ability to maintain cellular homeostasis, resulting in diminished tissue and cellular functions [26]. It is imperative to recognize that the disruption of proteostasis and the resulting buildup of unfolded and misfolded proteins are fundamental molecular characteristics of aging and various degenerative diseases [27, 28]. In mammals, the proteins and chaperones found in the ER lumen, which are crucial for preserving ER protein balance, such as protein disulfide isomerases (*PDI)*, binding immunoglobulin protein (*BiP)*, and calnexin, demonstrate reduced levels in different tissues of aging mice [29-33].

The diminished presence of chaperones in aging is a contributing factor to the accumulation and aggregation of unfolded proteins. Additionally, the increased oxidation rates experienced by these chaperones in aged cells result in structural modifications, ultimately leading to a deterioration in ER functionality within the cell [34]. The breakdown of the Unfolded Protein Response in aged organisms directly contributes to the accumulation of tissue damage and heightens susceptibility to disease [28]. Li and Holbrook [35] investigated the responsiveness of rat hepatocytes aged 24-26 months to a duo of ER stress agents, thapsigargin, and tunicamycin. Their results indicated that hepatocytes exhibit heightened susceptibility to ER stress as they age compared to younger cells. This heightened sensitivity was correlated with upregulated expression of the transcription factor C/EBP homologous protein (CHOP), indicating potential alterations in additional components of the UPR^ER^ signaling pathway during the aging process. Furthermore, rather than an adaptive UPR^ER^ response, there is a preference for an apoptotic pathway, a transition that is linked to modifications in proteostasis [34]. For example, aged mice demonstrate decreased expression of BiP and reduced phosphorylation of eIF2 in the cerebral cortex, which is associated with increased levels of GADD34 and pro-apoptotic proteins like activated caspase-12 [30]. In general, ProteinkinaseR-likeERkinase (*PERK*) [29, 36] and eukaryotic initiation factor 2 (*eIF2*) [30, 37-39] shows age-related changes in different tissues, while CHOP increases in older mice and rats [40, 41]. Concerning Immunoglobulin-Regulated Enhancer 1(*IRE1*)*/*X-box binding protein 1(*XBP1*) and *ATF6* are also modified with to age [33] but are tissue- and species-dependent, so the interpretation of it should be cautious and adjusted to the context in which the studies are conducted. Conversely, in the nematode C. elegans, a decrease in insulin/*IGF1*-like signaling has been associated with a notable extension in lifespan, a process that relies on the participation of IRE1 and XBP1. This implies that ER proteostasis might constitute an integral component of longevity [1]. The variations in protein content and resident chaperones within the ER during aging illustrate the various responses employed by aging cells to maintain homeostasis in this cellular compartment. Achieving homeostasis in the ER may provide insights into the role of this organelle in promoting longevity in older organisms. Specifically, organelles rely on proximity and communication to fulfill their functions and maintain cellular physiology [5]. As stated earlier, the interplay between mitochondria and the ER plays a crucial role in cellular responses to stress. This interaction is facilitated by physical connections, resulting in the formation of MAMs. These MAMs serve as pivotal sites for coordinating various cellular processes, such as calcium signaling, autophagy, lipid metabolism, apoptosis, and other functions [42]. The alterations in the composition of MAMs and the abnormal induction of ER-mitochondria association are significant factors in the development of age-related pathological conditions. The subsequent section will explore potential strategies for addressing these issues.

## Mitochondrial dynamics and MAMs

The malleable characteristics of mitochondria enable them to change morphology, structure, and abundance via fission and fusion mechanisms, essential for the preservation of proper physiological operations within both mitochondria and cells. The proximity between the ER and mitochondria, specifically when the ER encloses a segment of mitochondria, triggers mitochondrial fission. Studies by Lewis et al. demonstrated that the localized synthesis of mitochondrial DNA within the mitochondrial nucleoid of mammalian cells is linked to a limited number of stable ER-mitochondria contact regions. These sites coordinate mtDNA replication, facilitating and distributing newly replicated nucleoids to daughter mitochondria [43]. Key factors mediating mitochondrial fission include Dynamin-related protein 1 (*Drp1*), *Fis1*, and mitochondrial fission factor (*Mff*). *Drp1*, primarily located in the cytoplasm, is specifically recruited to ER-mitochondria contact sites by its receptors *Mff*, *Fis1*, and mitochondrial dynamics proteins 49 and 51 (*Mid49/51*) [44]. Drp1 assembles into helical oligomers at these locations, leading to the constriction and separation of membranes. inverted formin 2 (*INF2*), previously known to be present in the ER, was discovered to promote actin polymerization and assist in the recruitment of *Drp1* to contact sites between the ER and mitochondria. Interestingly, the absence of *Drp1* does not interfere with forming contact sites between the ER and mitochondria, indicating that *Drp1* may not directly link these organelles [45].

Mitochondrial fusion is vital in cardiac muscle cells, necessitating coordination between outer membrane fusion, mediated by *Mfn1/2*, and inner membrane fusion, regulated by *OPA1* [46, 47]. *Mff1* demonstrates higher GTPase activity than Mfn2 and can interact with *OPA1*. Additionally, *Mfn2* is occasionally found on the ER/Sarcoplasmic Reticulum and participates in ER/SR-mitochondria connections through physical interaction with *Mfn1* or *Mfn2* on the outer mitochondrial membrane [48]. Additionally, the process of mitochondrial fusion is modulated by endoplasmic reticulum-associated degradation (ERAD), a mechanism for quality control of proteins that directs ER proteins for degradation. Deficiency in ERAD results in a heightened proximity between mitochondria and the ER, elevated expression of *Sigma1R* in MAMs, and intensified interaction between *Mfn2* and other MAMs proteins, ultimately leading to excessive mitochondrial fusion through a mechanism that is not yet understood.

Mitochondrial fusion is essential in cardiac muscle cells, requiring coordination between outer membrane fusion (mediated by *Mfn1/2*) and inner membrane fusion (regulated by *OPA1*) [48]. *Mff1* demonstrates elevated GTPase activity compared to *Mfn2* and can engage with *OPA1*. In contrast, *Mfn2* is sporadically localized to the ER/SR and contributes to the connection between ER/SR and mitochondria by physically interacting with *Mfn1* or *Mfn2* on the outer mitochondrial membrane. Additionally, the process of mitochondrial fusion is governed by ERAD, a mechanism responsible for the degradation of ER proteins to maintain protein quality control.

## Lipid transport and synthesis at MAMs

MAMs play a crucial role in lipid metabolic processes and are essential for facilitating inter-organelle communication between the ER and mitochondria [49]. Phosphatidylserine (PS) is enzymatically synthesized in the ER by the enzymes *Pss1/2*. Following synthesis, PS is translocated into the mitochondria, which undergoes decarboxylation catalyzed by PS decarboxylase in the Inner Mitochondrial Membrane (IMM) to yield phosphatidylethanolamine (PE). PE is then promptly exported back to the ER, where it undergoes additional modifications to generate phosphatidylcholine (PC) through the action of the enzyme Phosphatidyl-ethanolamine N-methyltransferase (*PEMT2*) [50, 51]. Prior research indicates that the suppression of PSD activity results in a notable buildup of PS within MAMs [52]. The translocation of PS to mitochondria through MAMs is identified as the pivotal step in PE synthesis, thus serving as a crucial mechanism for regulating phospholipid equilibrium between the ER and mitochondria [53]. Moreover, mitochondria play a crucial role in synthesizing a unique lipid known as cardiolipin (CL), which is vital for mitochondrial bioenergetics and the initiation of apoptosis. The precursor phosphatidic acid (PA) required for CL synthesis is produced in the ER and subsequently transported into the mitochondria via the MAMs platform [54]. The mitochondrial-associated membrane is characterized by an abundance of lipid transport proteins and biosynthetic enzymes, such as Acyl-CoA synthetase long-chain family member 4 (*FACL4*), which facilitates the conjugation of fatty acids with cholesterol metabolites and coenzyme A [55], acyltransferase lyso-cardiolipin acyltransferase (*ALCAT1*)/Sterol O-acyltransferase 1 (*SOAT1*) is involved in cholesterol metabolism and transport [56], and Diacylgycerol Acyltransferase 2 (*DGAT2*) participates in the regulation of cholesterol esterification and triglyceride synthesis [57]. Therefore, it is imperative to investigate the processes through which proteins control lipid transport while also engaging in biosynthesis. As previously mentioned, MAMs consist of a complex comprising five subunits. Notably, murine double minute1 (*Mmm1*), murine double minute 2 (*Mdm12), and Mdm3* possess a Sterile Alpha Motif(SAM) and a Membrane Occupation and Recognition Nexus (MORN) domain, which are distinguished by the existence of a hydrophilic pocket in the phospholipid-lipid binding region [58]. The Mmm1-Mdm12 complex generates a continuous 210-Å-long hydrophobic tunnel that facilitates phospholipid transport [59]. Taken together, lipid metabolism is a key function of MAMs and is important for overall cellular health.

## Reactive Oxygen Species generation and MAMs

The collaboration of mitochondria, the ER, and MAMs in the generation of reactive oxygen species is a significant aspect to consider. Elevated levels of Reactive Oxygen Species (ROS) within cells and resulting oxidative harm to cellular components such as proteins, lipids, and DNA have been documented in various aging models [60-62]. While ROS serves as signaling molecules at moderate concentrations, excessive levels of these reactive species can be detrimental. The interactive nature of MAMs in facilitating communication between mitochondria and the ER suggests a potential role in modulating ROS production by these organelles [62, 63].

**Figure 2.**
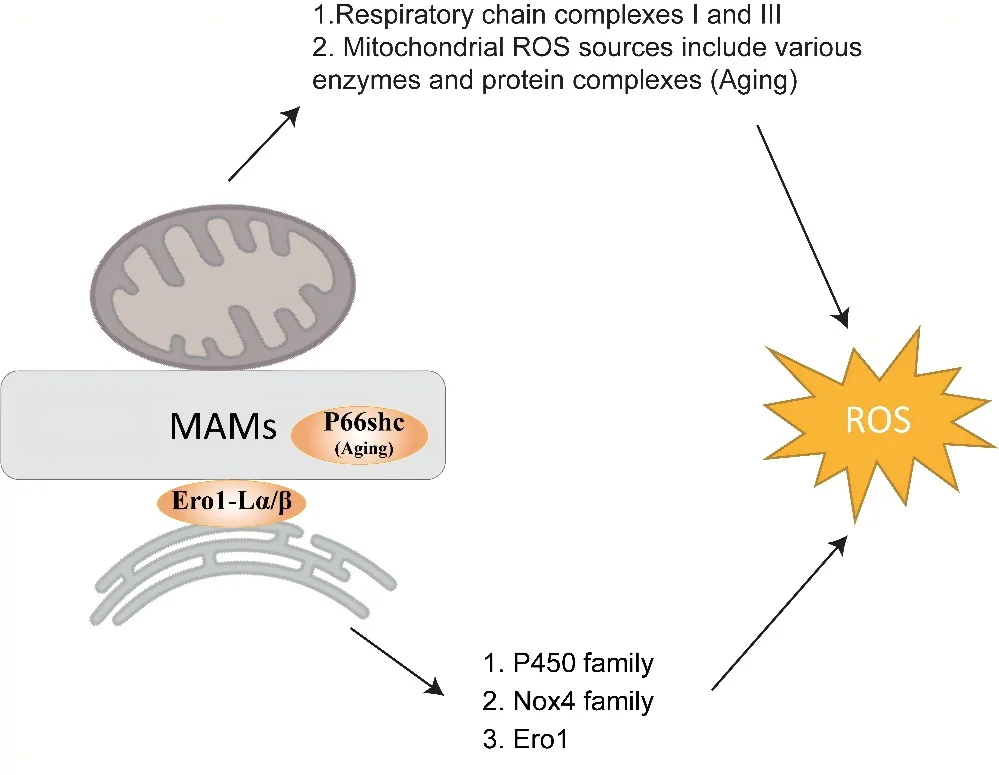
**Functionally, MAMs change with age, leading to increased oxidative stress and subsequent protein damage**. This includes alterations in the oxidative folding activity of the ER chaperone Ero-1α and electron transport by p66Shc at the mitochondrial ETC, which are linked to the development of age-related diseases.

The mitochondrial respiratory chain has been widely acknowledged as the predominant origin of harmful free radicals, such as O2•-, which are implicated in age-related oxidative stress [64, 65]. Nevertheless, there is a growing interest in other sources of intracellular reactive oxygen species, particularly enzymes situated in various subcellular regions [66]. The production of ROS within mitochondria primarily involves the generation of O2, which is subsequently converted to H_2_O_2_, leading to the formation of highly reactive OH [67]. Various components within mitochondria, such as respiratory chain complexes I and III, contribute to the generation of ROS [61]. Other known mitochondrial ROS sources include various enzymes and protein complexes, many of which are increasingly carbonylated during aging and senescence [68-71]. ROS production in the ER is a relatively understudied area, partly due to the limited availability of tools for accurately measuring ROS levels within this cellular compartment. However, certain proteins, such as members of the cytochrome P450 family [72], NADPH Oxidases (*Nox4*) [73], and endoplasmic reticulum oxidase 1 (*Ero1*) [74], have been identified as producers of ROS in the ER. Of particular note is *Ero1-Lα/β* [11, 75, 76], which localizes to the ER membrane, particularly in regions where MAMs are formed, suggesting a potential role of MAMs in ER-associated ROS production. Aging appears to be linked to an increase in oxidative damage and dysfunction of specific ER proteins, including the Ryanodine Receptors (RyR) [77] and chaperones such as protein disulfide isomerase and immunoglobulin heavy chain-binding protein [78, 79] (Fig. 2).

**Figure 3.**
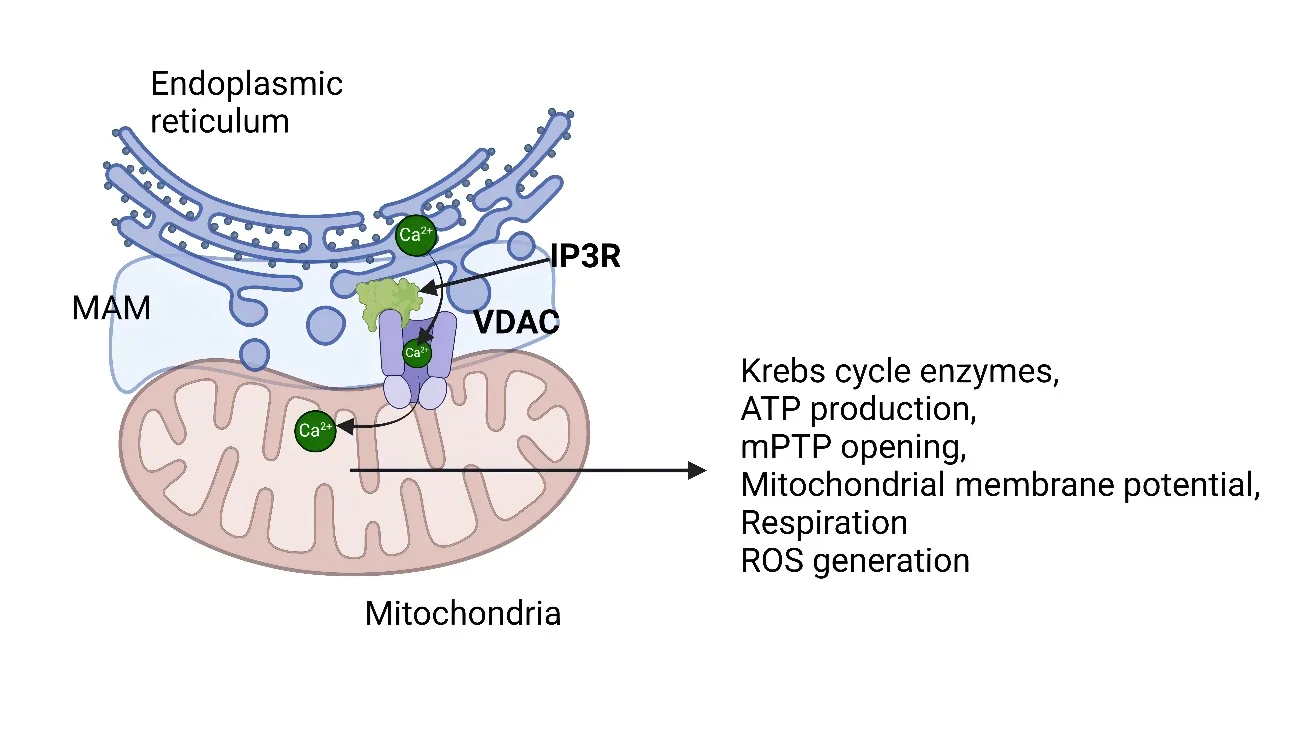
MAMs redox is responsible for ROS production: Ca^2+^ flux from the ER to MT through the IP3R/VADC Ca^2+^ channels, which generates redox nanodomain.

MAMs play a vital role in regulating ROS synthesis and are vulnerable to oxidative damage. The MAMs structure enables the transfer of calcium ions from the ER to the mitochondria by connecting Inositol 1,4,5-trisphosphate Receptor (*IP3R*) with voltage-dependent anion channel (*VDAC*) [80]. The influx of calcium ions into the mitochondrial matrix influences various aspects of mitochondrial function, such as the activity of Krebs cycle enzymes, Adenosine 5'-triphosphate (ATP) production, Mitochondrial permeability transition pore (mPTP) opening, mitochondrial membrane potential, respiration, and subsequently, ROS generation [81-84]. The relationship between ER function and mitochondrial ROS production is demonstrated by the decline in RyR function associated with aging [77, 83] (Fig. 3).

In aged skeletal muscle, the increased carbonylation and cysteine nitrosylation of RyR1 resulted in channel permeability, decreased calcium transients, heightened ROS levels, and compromised muscle contractile force. Furthermore, targeted overexpression of catalase in mitochondria attenuates oxidative alterations of RyR, while destabilization of RyR1 through rapamycin treatment exacerbates mitochondrial superoxide production. Furthermore, the mitigation of increased mitochondrial lipid peroxidation linked to RyR1 mutation-induced Ca^2+^ leakage through treatment with the antioxidant N-acetylcysteine suggests the involvement of ROS [85]. The translocation and enrichment of the MAMs fraction with the Ero1-Lα isoform are governed by the redox status of the ER environment [86]. Ero1-Lα, a FAD-dependent oxidase, works in conjunction with protein disulfide isomerase (*PDI*) in protein folding processes. PDI facilitates disulfide bond formation, while Ero1-Lα restores PDI's oxidized state by transferring electrons to molecular oxygen, resulting in the production of H_2_O_2_ synthesis [87, 88]. Additionally, Ero1-Lα modulates calcium release via MAMs and IP3R1 during ER stress, further emphasizing its role in ROS generation and calcium homeostasis regulation. Within the proteins present in MAMs, the 66-kilodalton isoform of the growth factor adapter-p66Shc protein has been associated with ROS synthesis and age-related illnesses. Belonging to the ShcA protein family, p66Shc functions as a dominant negative regulator in Ras-mediated signaling pathways and has been connected to oxidative stress and the aging process [89-91]. Deletion of p66Shc in mice results in increased resistance to oxidative and hypoxic stress, ultimately leading to a longer lifespan compared to animals with the wild-type gene [89].

The protein P66Shc, previously believed to be localized solely in the cytosol, has been observed in multiple cellular compartments such as the mitochondrial matrix, intermembrane space, outer mitochondrial membrane (OMM), and the mitochondria-associated membrane fraction. Phosphorylation of p66Shc at Ser36, triggered by both exogenous and endogenous oxidative stress, plays a crucial role in promoting its translocation to, or interaction with, mitochondria [89, 92]. Upon phosphorylation, p66Shc undergoes isomerization, dephosphorylation, and subsequent translocation to the MIMS and/or the MAMs fraction, where it plays a role in ROS generation [90, 92-95]. Within the MAMs, p66Shc facilitates the conversion of oxygen to H_2_O_2_ by consuming cytochrome c, a pivotal process in the initiation of apoptosis through the mitochondrial pathway [90]. The exact subcellular localization of p66Shc within mitochondria, specifically whether it resides in the MAMs or is attached to the OMM from the cytosolic side during the formation of MAMs, is a topic of ongoing discussion. Despite uncertainties regarding its precise cellular location, the involvement of p66Shc in the feedback loop of ROS-induced ROS production suggests its potential role in regulating mammalian lifespan. By converting oxidative stress damage into cellular apoptosis, p66Shc functions as an inducer of programmed cell death, thereby potentially reducing lifespan.

Additionally, research has demonstrated variations in the expression of p66Shc Messenger ribonucleic acid (mRNA) and protein across different cell types and tissues as individuals age [96]. Specifically, fibroblasts obtained from centenarians exhibited elevated levels of p66Shc expression compared to those from younger and older individuals. Conversely, primary cultures of skin fibroblasts derived from newborn and 18-month-old mice showed comparable levels of p66Shc [91]. Nevertheless, there was a notable increase in p66Shc expression in various tissues of adult mice compared to their newborn counterparts, suggesting age-related alterations in p66Shc expression [89]. Moreover, the elevated levels of p66Shc observed in the MAMs isolated from the livers of aged mice, along with the heightened production of ROS by mitochondria harboring MAMs, indicate the potential role of p66Shc in mediating the cellular response to oxidative stress associated with aging [91, 97]. Research conducted in MEFs has demonstrated that the absence of p66Shc ameliorates decreases in mitochondrial calcium signaling and changes in mitochondrial structure caused by oxidative stress, underscoring its significance in responding to oxidative stress and maintaining mitochondrial function [92]. The interplay between the ER and mitochondria, specifically via MAMs, is pivotal in the modulation of mitochondrial calcium uptake in senescent cells. Elevated transfer of Ca^2+^ from the ER to mitochondria in aged neurons cultured over extended periods is linked to cognitive decline associated with aging [98], potentially facilitated by increased expression of the MCU [99]. The regulation of calcium flux through MAMs entails the modulation of calcium channel expression levels, as well as the quantity and configuration of MAMs structures [100, 101]. Additionally, the ultrastructure of MAMs, specifically their thickness, plays a crucial role in determining the efficiency of calcium transport. Knockdown of Mitochondrial Calcium Uniporter (*MCU*) and Inositol 1,4,5-trisphosphate receptor type 2 (*ITPR2*) led to evasion of senescence [102], underscoring the significance of mitochondrial calcium accumulation in inducing senescence. Nevertheless, further investigation is necessary to elucidate the pivotal factors governing calcium fluxes through MAMs in aging cells.

The immune system plays a pivotal role in these resilience mechanisms. Notably, the aging process is characterized by chronic activation of the immune system, as evidenced by elevated levels of inflammatory markers in the bloodstream and the activation of immune cells both in circulation and within tissues. This phenomenon is referred to as "inflammaging." Similar to the aging process, inflammaging is linked to an elevated risk of numerous age-related pathologies and disabilities, in addition to frailty and mortality. In this paper, we examine recent advancements in the understanding of the mechanisms underlying inflammaging and the intrinsic dysregulation of immune function that accompanies aging.

The connection between the endoplasmic reticulum (ER)-mitochondria interface and inflammation was initially identified several years ago, following the observation that reactive oxygen species (ROS) facilitate the activation of NOD-like receptor protein 3 (*NLRP3*) inflammasomes [103]. The inflammasome is a multiprotein complex that includes a sensor protein, an adaptor protein known as apoptosis-associated speck-like protein containing a caspase-recruitment domain (*ASC*), and pro-caspase 1, a cysteine protease. Inflammasomes can be categorized into four subfamilies based on the sensor molecule involved: *NLRP3, NLRP1, NLRC4* (*NLR* family, *CARD* domain containing 4), and *AIM2* (absent in melanoma 2) [104]. These sensor molecules are capable of detecting a wide array of stimuli, including microbial products known as pathogen-associated molecular patterns (*PAMPs*), host-derived damage signals referred to as damage-associated molecular patterns (*DAMPs*), and various other cytosolic insults. Upon assembly, pro-caspase 1 undergoes autoprocessing through proximity-induced activation by caspase 1, leading to the maturation of prointerleukin-1β (pro-*IL-1β*) and pro-*IL-18* into their active forms. Among the various inflammasomes, *NLRP3* has been the most extensively studied and characterized, primarily due to its significant role in the pathogenesis of numerous human diseases [104]. As of now, the *NLRP3* complex remains the sole inflammasome complex identified in association with MAMs, despite extensive attention and investigation [105, 106]. Furthermore, various models have been proposed to elucidate the role of Ca^2+^ signaling in the activation of *NLRP3*. Notably, Lee et al. posited a pivotal role for the calcium-sensing receptor (*CASR*) in the activation of the *NLRP3* inflammasome, a process mediated by elevated intracellular Ca^2+^ levels and reduced cellular cyclic AMP (*cAMP*) [107]. An alternative model suggests that prolonged calcium ion influx, mediated through endoplasmic reticulum (ER) Ca^2+^ release channels, induces mitochondrial Ca^2+^ overload, subsequently leading to mitochondrial destabilization [107] and it is characterized by elevated levels of ROS production and the activation of the mitochondrial permeability transition pore (mPTP) [108]. In support of this model, Misawa et al. observed that during inflammasome formation, microtubules facilitate the perinuclear migration of mitochondria, leading to the subsequent juxtaposition of *ASC* on mitochondria with *NLRP3* on the endoplasmic reticulum [109].

## The commitment of MAMs and autophagy to lifespan regulation

In the quest for increased lifespan, cellular preservation from harm and demise is facilitated via autophagy, specifically macroautophagy, an essential catabolic mechanism responsible for breaking down and reusing cytosolic elements and organelles in reaction to cellular stress and metabolic requirements [110]. The development of the double-membraned autophagosome is a defining characteristic of autophagy, which then merges with lysosomes for the degradation of cargo by lysosomal enzymes [111]. Basal autophagy is vital for physiological quality control, yet its impairment is implicated in various human pathologies and aging [112]. There is ongoing discussion within the academic community concerning the specific membrane and lipid donor origins required for autophagosome assembly. Proposed sources include membranes derived from a variety of organelles such as mitochondria, the ER, golgi apparatus, plasma membrane, and more recently, mitochondria-ER contact sites [113]. Additionally, it has been observed that certain autophagy-related proteins, particularly Autophagy-related 5 (*ATG5*), show decreased levels in the brains of aging individuals [114]. Under starvation conditions, key proteins involved in AP formation, such as Double FYVE-containing protein 1 (*DFCP1*) and ATG14, relocalize toward the MAMs fraction, suggesting its role in autophagy regulation.

**Figure 4.**
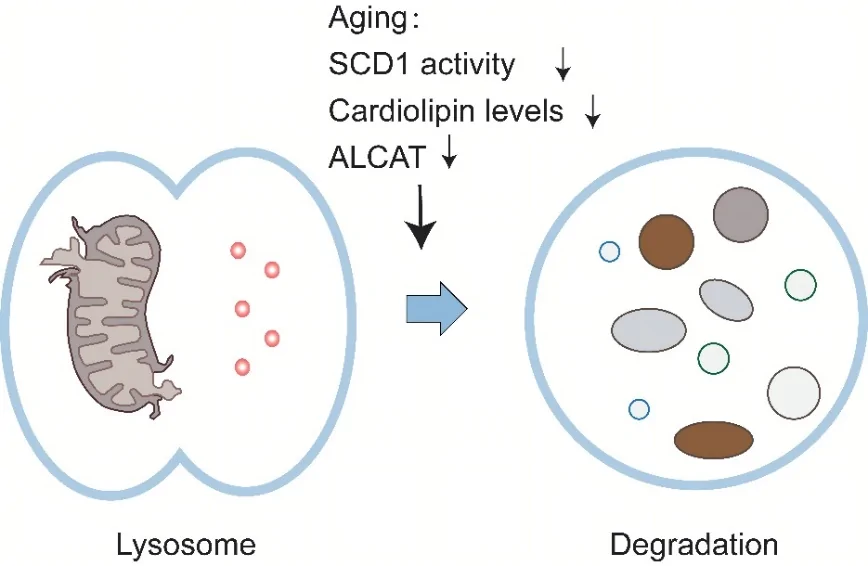
**MAMs emerged as important regulators of mitophagy/autophagy**. During mitophagy, the MAMs localized Mfn2 are phosphorylated by PINK1. Phosphorylated Mfn2 recruits parkin that, in turn, mediates Mfn2 ubiquitination leading to mitophagy initiation.

The impairment of MAMs integrity has been shown to hinder autophagosome formation in cells deficient in phosphofurin acidic cluster sorting protein 2 (*Pacs2*) and *Mfn2*, crucial proteins for MAMs functionality. Furthermore, disruption of MAMs through *Mfn2* depletion impedes the transfer of lipids between organelles and the initiation of autophagy. Levels of mitochondria-derived phosphatidylethanolamine and phosphatidylserine are correlated with longevity, underscoring the significance of lipid metabolism in the processes of aging and autophagy regulation. Lipid rafts, specifically gangliosides such as GD3, have been identified as playing a significant role in the biogenesis and maturation of autophagosomes, with GD3 being particularly abundant in the MAMs during autophagic induction. The level of membrane unsaturation has been linked to the aging process, with caloric restriction leading to a reduction in Polyunsaturated fatty acids (PUFA) content and subsequently decreasing vulnerability to peroxidative damage. Enzymes enriched in MAMs, such as Stearoyl-CoA Desaturase (*SCD1*), are involved in regulating the composition of cellular membranes, which in turn influences processes such as autophagy and aging. Reduced activity of SCD1 has been shown to impact autophagosome formation and the lipid composition of membranes. Changes in cardiolipin levels, an essential phospholipid delivered by the MAMs, are associated with the aging process, impacting the structure and function of the MAMs. The remodeling of cardiolipin, specifically by the MAMs-enriched enzyme acyl-CoA:lysoCL acyltransferase 1, plays a role in age-related diseases. Deficiency in ALCAT1 protects against conditions such as obesity, type 2 diabetes, and hepatosteatosis in mice, highlighting the importance of MAMs-mediated lipid metabolism in the regulation of aging and autophagy (Fig. 4).

## Function and dysfunction of MAMs in aging-related human diseases.

The process of aging is associated with an increased susceptibility to a range of disorders, including neurodegenerative conditions [115, 116], metabolic syndrome [117], cardiovascular diseases [60, 63, 118], and cancer [119].

## MAMs and neurodegenerative disorders

Notably, late-onset Alzheimer's and Parkinson's diseases (AD and PD) are among the most common age-related neurodegenerative disorders. Dysfunctional crosstalk between the ER and mitochondria is a prevalent characteristic in neurodegenerative diseases, as evidenced by various studies linking these pathologies to alterations in the structure and function of MAMs [120-123]. Additionally, a multitude of proteins implicated in neurodegenerative conditions have been identified in MAMs fractions, although the implications of their localization in MAMs are still being investigated [120, 124]. MAMs play a pivotal role in the pathogenesis of Alzheimer's disease by acting as the primary location for the production of β-amyloid peptide [125]. The release of Aβ takes place at MAMs during the processing of the amyloid precursor protein by the γ-secretase complex, which comprises Presenilin 1 and Presenilin 2.

In genetic forms of Alzheimer's disease, aberrant Presenilin 2 proteins have been shown to interfere with ER-mitochondria connections and their associated physiological processes [121]. Comparable disruptions have been documented in amyloid precursor protein transgenic mouse models and neuronal cells exposed to amyloid-β [126]. Notably, the presence of AD-related proteins at MAMs can elicit conflicting outcomes, with certain instances demonstrating a marked enhancement in MAMs functionality and abundance in response to mutant AD proteins [122, 127]. Interestingly, the e4 allele of ApoE4, a prevalent risk factor for Alzheimer's disease-related senile dementia, has been demonstrated to enhance the activity of MAMs [128]. Conversely, in certain instances, Alzheimer's disease proteins lead to a reduction in ER-mitochondria tethering [126]. Similarly, proteins associated with Parkinson's disease such as α-synuclein, Parkin, and *DJ-1* have been found to facilitate ER-mitochondria connections [122-124, 127]. For example, α-synuclein is present in MAMs [124] fractions where it forms stable interactions with lipid raft domains of mitochondrial membranes. The impact of α-synuclein mutations on the structure of MAMs is heterogeneous; some studies report a reduction in ER-mitochondria contacts, while others observe an increase in the number of MAMs in Parkinson's disease cells [122]. The diverse responses of MAMs to proteins associated with Alzheimer's or Parkinson's disease highlight the necessity for further investigation to elucidate the molecular mechanisms underlying MAMs function in various pathological contexts.

ER stress is a prevalent characteristic observed in neurodegenerative disorders such as Alzheimer's disease, Parkinson's disease, Huntington's disease, and amyotrophic lateral sclerosis [129]. Various factoon referred to as the UPR, which encompasses three interconnected pathways: *PERK, IRE1*, and *ATF6* [130]. The pathways under consideration are designed to either restore rs can trigger the buildup of misfolded proteins within the ER, resulting in ER stress [131, 132]. This prompts an adaptive reactiproteostasis or, in instances of failed recovery, trigger cell death. Research findings indicate a significant correlation between the ER stress response and neurodegenerative diseases, underscoring the potential of the unfolded protein response pathways as viable targets for therapeutic intervention [129]. Notably, multiple studies have highlighted a strong connection between MAMs, ER stress, and the unfolded protein response.

Numerous studies have highlighted the role of MAMs tethering factors, specifically *Mfn-2* and V protein-associated protein B (*VAPB*), in facilitating ER-Mitochondria connections. Furthermore, MAMs are important sites for essential ER chaperones, including Glucose-Regulated Protein 78 (*GRP78*)*/*binding immunoglobulin protein (*BiP)*, *calnexin*, *calreticulin*, Endoplasmic reticulum resident protein (*ERp44*), *ERp57*, and the *Sigma 1* receptor. Interestingly, MAMs also contain UPR transducers such as *IRE1* and *PERK*. At MAMs, *IRE1* interacts with *Sig1R* to enhance ER-to-mitochondria survival pathways [133, 134]. Additionally, at MAMs, *PERK* serves as both a tethering factor and a regulator of ROS transport, thereby impacting the propagation of apoptotic signaling [135]. Notably, the activity of *PERK* is modulated by the MAMs tethering factor *Mfn2* [136]. In recent years, a multitude of compounds have been discovered for their ability to regulate ER stress, however, their impact on MAMs remains largely uninvestigated [129]. As previously mentioned, the *PERK* signaling pathway is frequently hyperactive in numerous neurodegenerative disorders, and this aberration is linked to neuronal apoptosis in both Alzheimer's disease and Parkinson's disease models.

Consequently, the inhibition of PERK signaling through the use of targeted *PERK* inhibitors, such as GSK2606414, has demonstrated neuroprotective properties [137]. It is noteworthy that in Alzheimer's disease, the activation of *PERK* is linked to heightened memory decline and β-amyloidogenesis, which takes place at the interface of the ER and mitochondria. Importantly, studies utilizing in vivo AD models have shown that memory deficits can be reversed by suppressing PERK expression [138]. Similarly, research has demonstrated that genetic or chemical inhibition of IRE1 signaling can play a protective role in Alzheimer's disease by decreasing the expression of amyloid precursor protein and subsequent β-amyloid deposition [139]. Additionally, studies have shown that inhibition of *PERK* can reduce *Mfn* contacts at the ER-Mitochondria interface. In a recent study involving an early-onset Parkinson's disease model (PARK20), where *PERK* is consistently activated, inhibition of *PERK* phosphorylation by GSK2606414 has been found to have positive effects on ER stress and mitochondrial dysfunction [140]. The results of Celardo et al. [141] demonstrate that in Parkinson's disease models with pink1 and parkin mutations, inhibition of *PERK* signaling leads to neuroprotective effects by decreasing *Mfn* interactions with the ER, thereby enhancing ER stress signaling. These findings provide compelling evidence for the potential benefits of targeting ER stress reduction as a therapeutic strategy for neurodegenerative diseases, highlighting the importance of further research to identify optimal molecular targets within MAMs components. Metabolic syndrome, a condition associated with aging, affects around 20% of the elderly population in developed nations.

## MAMs, Metabolic syndrome and cardiovascular diseases

Metabolic syndrome is defined by the co-occurrence of various cardiometabolic risk factors including obesity, insulin resistance, dyslipidemia, and hypertension [142]. Numerous studies have examined the impact of these risk factors on ER-mitochondria membrane connections. In different scenarios, the organization and operation of MAMs display varied reactions. For example, in animal models of obesity, there is an observed rise in the quantity of MAMs within hepatic cells, causing an overload of mitochondrial Ca^2+^ and subsequent dysfunction of the mitochondria [143]. Conversely, in hypothalamic cells, the reduction of MAMs through the elimination of the tethering factor Mfn2 leads to leptin resistance induced by ER stress, increased food intake, decreased energy expenditure, and ultimately the development of obesity [144]. Insulin is essential in the aging process, with the maintenance of the structural integrity of MAMs being critical for the effective functioning of insulin signaling pathways [145]. Disruption of MAMs structural components hinder insulin signaling, whereas increased expression of MAMs proteins improves insulin signaling. The restoration of insulin sensitivity through pharmacological means reestablishes the connections between the ER and mitochondria, underscoring the reciprocal regulation between these biological processes [145].

Furthermore, it has been observed that ER stress plays a role in the impairment of insulin synthesis in pancreatic β cells and the development of insulin resistance. This suggests that targeting the ER stress pathway through pharmacological intervention could potentially improve insulin deficiency [146]. Cardiovascular diseases are commonly linked to metabolic syndrome, particularly conditions such as obesity, insulin resistance, and type 2 diabetes. Within cardiomyocytes, MAMs regulate the uptake of mitochondrial Ca^2+^, which is crucial for insulin signaling. Disruptions in this process have been associated with cardiac hypertrophy[147, 148]. Deficiency of the MAMs tethering factor Mfn-2 in patients or animal models of pulmonary arterial hypertension disrupts ER-Mitohcondria membrane contacts and contributes to pulmonary artery smooth muscle cell hyperproliferation [149].

Endothelial dysfunction frequently manifests in the elderly population and is closely linked to hypertension and other cardiovascular diseases, often ascribed to increased oxidative stress. The development of cardiovascular diseases resulting from endothelial dysfunction involves a complex interaction of ER stress, the unfolded protein response, and oxidative stress. This indicates a potential opportunity for therapeutic interventions aimed at these pathways in older individuals [150]. In numerous cell types found in the cardiovascular system, such as cardiomyocytes, endothelial cells, and vascular smooth muscle cells, there is a significant presence of ER, mitochondria, and mitochondria-associated membrane microdomains. Mitochondria play a crucial role in the regulation of cellular metabolism, ATP production, ROS generation, and cellular apoptosis. MAMs actively participate in regulating cell signaling, particularly calcium, ROS, and lipid signaling, as well as membrane dynamics, lipid metabolism, autophagy, and apoptosis processes. Consequently, they exert critical regulatory roles in maintaining mitochondrial physiological functions [151].

As individuals age, cells in various tissues may experience mitochondrial dysfunction and ER stress as a result of detrimental environmental influences. This can lead to impaired mitochondrial calcium uptake, heightened mitochondrial fission, compromised autophagic activity, buildup of mutated mitochondrial DNA (mtDNA), and aberrant lipid transport across MAMs. Changes in the molecular makeup and operation of MAMs are intricately associated with the initiation and advancement of cardiovascular ailments [151].

In cardiomyocytes, the regulation of sarcoplasmic reticulum calcium is mediated by the *MCU* located in the MAMs. With advancing age, there is a decrease in the expression of *MCU* and its regulatory subunit (*MICU1*) within the MAMs, leading to compromised calcium uptake by the mitochondria. Consequently, this dysregulation of calcium homeostasis contributes to the development of diastolic dysfunction in the myocardium [152]. In addition, Sharov et al. identified age-related post-translational modifications, including oxidation and nitration, of Sarco/Endoplasmic Reticulum Calcium ATPase 2 (*SERCA2*) in aging cardiac tissues. These alterations lead to a marked decrease in SERCA2 activity, thereby playing a role in the development of age-related diastolic dysfunction [153]. Therefore, abnormalities in MAMs-mediated calcium regulation may accelerate susceptibility to cardiovascular diseases.

The regulation of mitochondrial dynamics is impacted by the energy requirements and availability of nutrients, potentially functioning as a mechanism for biological energy adaptation in the context of cardiac pathological remodeling [154]. An overabundance of mitochondrial fission has been identified as a substantial contributor to the aging process. The depletion of mitochondrial fusion proteins Mfn1 and Mfn2 has been shown to expedite aging and trigger mutations in mitochondrial DNA [155]. On the contrary, upregulation of mitochondrial fission proteins, specifically *Drp1* and *Fis1*, induces mitochondrial fragmentation, initiates the autophagy-lysosome-proteasome pathway, and ultimately results in cardiomyocyte injury and depletion [156]. Moreover, the role of mitochondrial-mediated autophagy in cellular quality control is significant. The decline in autophagy has been linked to an increased vulnerability to cardiovascular diseases in the aging population. Considering that most adult cardiomyocytes are terminally differentiated and have limited ability to proliferate, a strong autophagy process is essential for maintaining the functionality and structural balance of the aging heart [157]. The decline in expression levels of autophagy-related proteins, such as *Binp3* and *PARKIN*, with advancing age results in compromised removal of misfolded proteins and dysfunctional organelles [158]. During the process of aging, a notable reduction in the mRNA expression levels of autophagy markers *ATG5* and *BECN1* in the cardiac tissue signifies a decrease in autophagy activity. Furthermore, there is an observed decline in *PARKIN*-mediated mitochondrial autophagy in the hearts of aging mice. It is noteworthy that the upregulation of *PARKIN* in cardiomyocytes has been demonstrated to delay the onset of age-related cardiac dysfunction, thereby proposing a potential therapeutic approach for alleviating age-related cardiovascular deterioration.

Research by Zou et al [159]. indicates that FUN14 domain containing 1(*FUNDC1*), located in the MAMs region, can bind to Inositol 1,4,5-trisphosphate Receptor (*IP3R2*) and mediate the release of Ca^2+^ from the ER into the mitochondria and cytoplasm of cardiomyocytes. The disruption of MAMs structure due to the loss of *FUNDC1* results in decreased expression of *IP3R2* in cardiomyocytes, leading to diminished levels of mitochondrial and cytoplasmic Ca^2+^. The decrease in Ca^2+^ levels hinder the expression of Fis1, a Ca^2+^-sensitive transcription factor regulated by CAMP -response element binding protein (CREB), thereby obstructing mitochondrial fission and compromising its functionality. Consequently, this series of events culminates in cardiac dysfunction and ultimately heart failure [159]. Increased levels of IP3 in mature cardiomyocytes are frequently linked to the progression of myocardial hypertrophy, arrhythmias, and heart failure. Seidlmayer et al. illustrated that following the activation of cardiomyocytes with isoproterenol, the release of Ca^2+^ from the sarcoplasmic reticulum through the RyR pathway is enhanced, primarily through the promotion of RyR phosphorylation and augmentation of SR Ca^2+^ leakage. This Ca^2+^ subsequently migrates into the mitochondria to activate the *MCU*, facilitating the influx of Ca^2+^ into the mitochondrial matrix.

On the contrary, the activation of cardiomyocytes by endothelin-1 results in an elevation of intracellular IP3 levels, leading to the initiation of the *IP3R*-mediated Ca^2+^ release pathway. Subsequently, these Ca^2+^ ions trigger the opening of mRyR1 channels and are subsequently sequestered by the mitochondrial matrix. This observation implies that, in normal physiological circumstances, the *IP3*-mRyR1 pathway may function as a supplementary mechanism for sustaining mitochondrial respiration and ATP production [160]. Mitochondria, which are integral to ATP production and cellular energy metabolism, frequently demonstrate malfunction in cases of heart failure. Malfunctioning mitochondria are a significant contributor to the generation of ROS, which have the potential to induce cardiomyocyte apoptosis and necrosis. Mitochondrial autophagy, also known as mitophagy, is essential for the degradation of dysfunctional mitochondria.

Shirakabe et al. illustrated that mitochondrial autophagy undergoes transient activation at multiple time intervals during the three-day period subsequent to Transverse Aortic Constriction (TAC) surgery in murine cardiac tissue, concomitant with an elevation in *Drp1* expression within the mitochondria. Nevertheless, by the fifth day post-TAC surgery, there is a reduction in mitochondrial *Drp1* levels, concomitant with a decrease in mitochondrial autophagy. The downregulation of mitochondrial autophagy is a significant factor in the pathogenesis of heart failure, as it contributes to mitochondrial dysfunction. Enhancing mitochondrial autophagy may offer a promising strategy for ameliorating both mitochondrial dysfunction and heart failure [161].

Mitochondrial ROS regulates changes in mitochondrial dynamics, mitochondrial autophagy, and post-translational modifications during Myocardial Ischemia-Reperfusion Injury (MIRI), thereby playing a pivotal role in the pathophysiology of MIRI [162]. During ischemia/reperfusion, the activation of soluble adenylyl cyclase 10 leads to the accumulation of *cAMP* and the activation of Mitochondrial Exchange Protein directly activated by *cAMP 1* (Epac1), also known as MitEpac1. Through the modulation of the *VDAC1-GRP75-IP3R1* complex, MitEpac1 facilitates the translocation of Ca^2+^ from the ER to the mitochondria, resulting in mitochondrial Ca^2+^ overload, increased generation of ROS, and the induction of mitochondrial permeability transition pore opening, ultimately resulting in the demise of cardiomyocytes.

Moreover, MitEpac1 facilitates the recruitment of Calcium/Calmodulin-Dependent Protein Kinase II (*CaMKII*) and Isocitrate Dehydrogenase 2 (*IDH2*) to assemble a protein complex within the mitochondria, resulting in the inhibition of IDH2 activity and subsequent reduction in Triphosphopyridine nucleotide (NADPH) synthesis. Consequently, this diminishes the antioxidant capacity of cardiomyocytes [162]. Zheng et al. illustrated that *Nogo-B* expression is elevated in murine hearts afflicted with myocardial infarction and in cardiac microvascular endothelial cells subjected to hypoxic and glucose-deficient environments. Enhanced *Nogo-B* levels in endothelial cells stimulate the *Notch1* signaling cascade, resulting in the upregulation of *Hes1* in infarcted hearts. This process fosters network development in cardiac microvascular endothelial cells, thereby enhancing collateral vessel formation. Consequently, this phenomenon diminishes the size of myocardial infarctions and ameliorates cardiac function [163]. The zinc transporter zinc transporter 7 precursor (*ZIP7*), which plays a crucial role in regulating Zn^2+^ concentrations within mitochondria, exhibits increased expression in the cardiac tissues of individuals with heart failure and in murine hearts exposed to ischemia/reperfusion injury. This heightened expression of *ZIP7* contributes to a reduction in Zn^2+^ levels within mitochondria, leading to mitochondrial hyperpolarization. Furthermore, it hinders the localization of *PINK1* and *PARKIN* to mitochondria, thereby impeding mitochondrial autophagy and exacerbating myocardial infarction.

On the contrary, the knockout of *ZIP7* results in mitochondrial depolarization through the elevation of Zn^2+^ concentrations, leading to the enhanced localization of *PINK1* and *PARKIN* within mitochondria and subsequently promoting mitochondrial autophagy. This process ultimately mitigates mitochondrial ROS production and ameliorates myocardial infarction [164]. The absence of *Mfn2*, a crucial protein in the MAMs, hinders the communication between cardiomyocyte mitochondria and the ER. This impediment diminishes the translocation of calcium ions from the ER to mitochondria, thereby shielding mitochondria from the detrimental effects of calcium overload during episodes of acute myocardial ischemia/reperfusion injury. Consequently, the prevention of mitochondrial calcium overload results in a decrease in ROS generation, ultimately ameliorating the extent of myocardial infarction [165]. Studies have demonstrated that the glycogen synthase kinase-3β (*GSK3β*) protein in cardiac tissue exhibits a specific binding affinity and interaction with *IP3Rs* located in the MAMs region. Upon induction of ischemia/reperfusion, the activation of *GSK3β* and phosphorylation of IP3Rs result in an elevation of Ca^2+^ transfer from the sarcoplasmic reticulum to the mitochondria. Consequently, the inhibition of *GSK3β* during ischemia/reperfusion has been shown to significantly mitigate the release of Ca^2+^ from sarcoplasmic reticulum *IP3R* channels, alleviate both cytoplasmic and mitochondrial Ca^2+^ overload, and ultimately confer protection to cardiomyocytes [166]. During ischemia/reperfusion injury, the dephosphorylation of *Drp1* at serine 637 (*Drp1S637*) induces the mitochondrial translocation of *Drp1*, thereby promoting heightened mitochondrial fission, elevated levels of ROS, and an overload of calcium. The inhibition of *Drp1*, whether administered prior to or following ischemia/reperfusion, can effectively diminish left ventricular end-diastolic pressure and mitigate the extent of ischemia/reperfusion injury [167].

## MAMs and Cancer

The age-related decline in organ function is a significant contributing factor to the development of cancer. Cancer cells demonstrate changes in protein expression that impact the functionality of MAMs, specifically in the transport of calcium ions between the ER and mitochondria. This alteration in MAMs function contributes to cancer cell characteristics such as resistance to programmed cell death, uncontrolled cell growth, increased ability to spread to other tissues, and changes in cellular metabolism [168, 169].

The aberrant expression of proteins localized to the ER-mitochondria interface in cancer has been extensively documented, with the sigma-1 receptor being notably upregulated in cancer cells relative to healthy tissues [170, 171]. As previously mentioned, the IP3R protein functions as a pivotal regulator in mediating the transfer of calcium ions from the ER to the mitochondria and is implicated in the fundamental mechanisms of tumor formation in diverse types of cancer. Of the three identified isoforms of *IP3R (IP3R1-3)*, research has shown that *IP3R1* and *IP3R2* are upregulated in both healthy and colon cancer tissues. On the contrary, IP3R1 has exclusively been identified within intracellular pathways in cancer cells, with its expression being associated with cell invasion and metastasis. Suppression of intracellular *IP3R3* has been observed to lead to elevated levels of apoptosis in ovarian cancer A2780 and colorectal cancer DLD1 cell lines [172].

*SERCA* channels located in the ER are the main mediators of calcium uptake. The activity of these channels is modulated by oncogenes and tumor suppressors present in the MAM, consequently impacting cancer cell survival through the modulation of ER calcium homeostasis [173]. The tumor suppressor gene *p53*, which is commonly mutated in approximately 50% of aggressive tumors, has been shown in a previous study to exhibit reduced expression in human colon cancer HCT-116 cells. This decreased expression is associated with a notable decrease in ER Ca^2+^ uptake and a diminished capacity for Ca^2+^ to be transported to the mitochondria [174]. Following treatment of cancer cells with adriamycin, a chemotherapeutic agent, the tumor suppressor protein p53 is activated and localizes to the MAMs. Subsequently, *p53* interacts with the *SERCA* pump, mitigating its oxidation and enhancing its function. This cascade of events culminates in mitochondrial calcium overload, triggering the release of caspase cofactors that facilitate programmed cell death, or apoptosis.

Additionally, Thioredoxin-Related Transmembrane Proteins (*TMX1*), another candidate gene, is found to be enriched in MAMs and has the ability to inhibit the function of *SERCA2b* [100]. The findings of an experimental study indicate that melanoma cells (A375P) with decreased *TMX1* expression exhibit elevated ER-Ca^2+^ levels and decreased translocation of Ca^2+^ to mitochondria. These results suggest that reduced *TMX1* levels may redirect cellular bioenergy away from mitochondria by attenuating ER-mitochondrial interactions, thereby promoting tumor progression [175]. In summary, the dysregulation of calcium ion homeostasis in the tumor microenvironment is intricately associated with the transport of Ca^2+^ from the ER to mitochondria. This transport process is governed by Ca^2+^ transporter proteins, tumor suppressors, and oncogenes. Disruption of Ca^2+^ homeostasis, triggered by diverse factors, can either facilitate cell apoptosis or survival, consequently impacting tumorigenesis. Moreover, MAMs play a crucial role in tumorigenesis by providing a platform for the dysregulation of Ca^2+^ homeostasis. Oncogenes, including Myeloid cell leukemia-1 (*Mcl-1*), B-cell lymphoma-2 (*Bcl-2*), serve to inhibit mitochondrial Ca^2+^ overload and enhance the viability of cancer cells, whereas tumor suppressor genes such as *PTEN* and Breast cancer susceptibility gene (*BRCA2*) facilitate ER-mitochondria Ca^2+^ transfer and trigger apoptosis in cancer cells [168, 176].

Members of the Bcl-2 family, particularly Bcl-2 and *Bcl-xl*, situated in the ER, play a crucial role in modulating *IP3R* activity and apoptosis by regulating the release of cytochrome C and the activation of cysteine aspartate proteases. In the context of ovarian cancer, downregulation of *Bcl-2* facilitates the enhanced transport of calcium ions from the ER to mitochondria via MAMs. This process results in mitochondrial calcium overload, leading to an upregulation of calpain-1-mediated apoptosis [175]. Moreover, the upregulation of the *Bcl-2* gene has been observed in non-small cell lung cancer, providing a protective mechanism for cancer cells against apoptosis through the sequestration of pro-apoptotic family members and the modulation of *IP3R*-mediated calcium ion signaling. Conversely, *Bcl-xl* demonstrates a sensitizing influence on *IP3Rs* [177]. *Bcl-xl* has been found to suppress *IP3R*-mediated Ca^2+^ release through interactions with the same IP3R region as *Bcl-2*. In multiple cell systems, including breast cancer cells, *Bcl-xl* inhibits Ca^2+^ release from the ER to the cytoplasm, thereby providing protection against IP3R-mediated apoptosis [178]. Furthermore, *Bcl-xl* can suppress *VDAC1*-mediated mitochondrial Ca^2+^ uptake in breast cancer cells, leading to changes in mitochondrial ATP generation and increased ROS production, ultimately promoting cancer cell migration [179].

MAMs have been identified as beneficial locations for a range of anti-cancer treatments aimed at disrupting oncogene activity or enhancing ER-mitochondrial calcium transfer, ultimately increasing the susceptibility of cancer cells to apoptosis or impeding pro-tumorigenic processes [180, 181]. In summary, MAMs are crucial in the modulation of numerous functions disrupted in age-related illnesses. Further investigation of MAMs alterations in these disorders holds promise for discovering novel therapeutic targets to restore correct ER-Mitochondria interplay and prevent the development of pathological features.

## Dietary interventions have an impact on MAMs Lipids play a part in MAMs

Sphingolipids function as membrane components and signaling molecules and play a critical role in human disease. The dihydroceramide desaturase Δ4-dihydroceramide desaturase 1 (*DEGS1*) acts as part of the sphingolipid synthesis pathway, i.e., de novo ceramide biosynthesis. DEGS1 is a mitochondria-associated endoplasmic reticulum membrane-resident (MAMs-resident) enzyme, and *DEGS1* deficiency has been reported to disrupt major core MAMs functions [182]. Dietary intake of *MFGM/EV*-enriched concentrates promotes the accumulation of very long, odd-chained sphingolipids and increases lipid metabolism turnover at the systemic level [183]. The *MFGM/EV* enriched dietary intake of *MFGM/EV* concentrates promotes accumulation of very long odd-chained sphingolipids and increases lipid metabolic turnover at the systemic level. However, in obese, overweight populations, ramadan diurnal intermittent fasting is associated with decreased plasma sphingomyelin and sphingomyelin, whereas decreased sphingomyelin 1-phosphate and sphingosine 1-phosphate are accompanied by decreased sphingomyelin 1-phosphate, and RIF is associated with decreased C17, C22, and C24 sphingolipids [184].

High-fat diet affects the structure and function of MAMs in several models of aging-related diseases. A high-fat diet in humanized resistin mice increased fragmented and shorter mitochondria in skeletal muscle, whereas resistin-KO mice were found to have healthy normal mitochondria. The resistin-induced mitochondrial fission was accompanied by an increase in MAMs formation. Moreover, the resistin-*CAP1* complex may be a potential therapeutic target for the treatment of obesity-related metabolic diseases, such as diabetes and cardiometabolic diseases [185]. High fat diet induced mitochondrial fission in aortic tissues of HFD mice contributes to atherosclerosis [186]. High fat diet rich in saturated fatty acids (high lard, HL, diet) diet in the liver caused a decrease in *Mfn2* and an increase in proteins involved in the fission process (*Drp1* and *Fis1*), accompanied by many small round mitochondria. Similarly, weak signals for fusion proteins and strong signals for fission proteins were shown in skeletal muscle of rats fed the HL diet [187]. In addition, saturated fatty acids have been reported to induce the fission process in differentiated *C2C12* skeletal muscle cells associated with mitochondrial dysfunction in vitro [188]. Higher fat/carbohydrate ratios or diets increasing marine fatty acids have not shown an effect on mitochondrial fusion, fission in mouse intestinal epithelial cells significantly 29962969. Abnormal mitochondrial fusion and fragmentation can affect MAMs structure and function in a variety of aging-related disease models [189, 190].

For example, *Mfn2* has different effects on MAM regulation in different conditions and cell types [191-195]. Furthermore, in the direction of tumor cardiology research, sorafenib (sor)-mediated *Mfn2* downregulation in a concentration-dependent manner was found. Furthermore, reduced mitofusin-2 level augmented sor-mediated elevated MAMs biogenesis and increased mitochondria-MAMs tethering in cardiomyocytes [196]. The above results suggest that the HL diet affects MAMs structure and function by promoting mitochondrial fission and decreasing mitochondrial fusion, thereby influencing disease outcome and drug efficacy.

Omega 3 polyunsaturated fatty acids have been reported to improve mitochondrial function and promote mitochondrial fusion compared to the effects of saturated fatty acids. In an in vitro model of steatotic hepatocytes, both eicosapentaenoic acid and docosahexaenoic acid increased the expression of *Mfn2* and ATP levels. In contrast, in *Mfn2*-depleted steatotic hepatocytes, omega 3 PUFA did not induce the previously described effects [197, 198]. The shift toward fusion was found to be accompanied by an increase in *Mfn2* and *OPA1* and a decrease in *Drp1* and *Fis1* in the livers of high-fat diet fish oil-rich (HFO diet) HFO diet-fed rats, and this fusion phenotype was consistent with a decrease in body weight gain in both HFO diet and HL diet-fed rats [199]. Skeletal muscle from HFO-fed rats showed more immunoreactive fibers for *Mfn2* and *OPA1* proteins and weaker immunostaining for *Drp1* and *Fis1* compared to HL-fed rats. (Lionetti et al., 2013) (doi: 10.4236/fns.2013. 49A1017) [198]. Abnormalities in mitochondrial fusion and fission have been described previously as affecting MAMs structure and function in a variety of aging-related disease models [189, 190]. These suggest that an unsaturated fatty acid diet affects MAMs structure, and function by affecting mitochondrial fission and fusion.

## Effect of ketone body diet on MAMs

The ketogenic diet improved ischemic tolerance and mitochondrial function in the brain by preventing *Drp1*-mediated mitochondrial fission and activation of *NLRP3* inflammatory vesicles, thus providing neuroprotection [200]. The ketogenic diet also enhances the efficacy of chemotherapy and radiotherapy by promoting mitochondrial fission, suggesting its potential in cancer therapy [201]. And as previously described, abnormal mitochondrial fusion and fission affects the structure and function of MAMs in multiple models of aging-related diseases [189, 190]. The above results suggest that the ketogenic diet affects MAMs structure, function, and thus disease regression by influencing mitochondrial fission.

## Effect of starvation, caloric restriction on MAMs

Mitochondrial dynamics behave differently in two different nutritional deficiency conditions, such as starvation [202] and caloric restriction (CR) [203]. Either glucose or serum elimination increases mitochondrial fragmentation, whereas mitochondrial fusion is induced by nitrogen source deficiency (glutamine or amino acids). It is suggested that mitochondrial dynamics can be regulated according to the type and severity of starvation. Starvation-induced mitochondrial fusion is dependent on *Mfn1* and *OPA1* and is mediated by decreasing *Drp1* fission activity and preventing *Drp1* from localizing to the mitochondria [198]. Dynamic observation of mitochondria in the CR animal model showed an increase in proteins associated with mitochondrial fission (*Fis1* and mitochondrial *Drp1*) but no detectable proteins involved in mitochondrial fusion (*Mfn1* /*Mfn2* and *OPA1*) were changed [202]. A significant increase in the number of mitochondria per cell as well as parameters related to mitochondrial biogenesis was also found under CR conditions [198, 204, 205]. This suggests that different nutrient-deficiency conditions lead to different MAMs effects by having different effects on mitochondrial dynamics.

During the metabolic transition from fasting to feeding, MAMs integrity is disrupted, which induces mitochondrial fission and reduces mitochondrial cristae density in human peripheral blood mononuclear cells (PBMCs) [206, 207]. In addition, fasting or calorie restriction increases the abundance of the ketone body d-β-hydroxybutyrate (βOHB), which is a class I histone deacetylase (HDAC). endogenous and specific inhibitor of histone deacetylase [208]. Reducing class I histone deacetylases (e.g., HDAC2 and HDAC3) reduces the number of MAMs [209].

## Concluding remarks

In the current biological perspective, the direct connection between the molecular composition of MAMs and aging remains largely overlooked and requires further scientific investigation. However, indirect evidence suggests that MAMs significantly influences cellular function and longevity:

- The gradual decrease in mitochondrial calcium uptake with cell passage and the reduced number of MAMs in senescent cells imply a role for MAMs in aging-related cellular changes.
- The correlation between the abundance of p66Shc protein in MAMs and animal lifespan indicates a potential link between MAM function and longevity.
- MAMs play a crucial role in regulating lipid fluxes and autophagy, both of which are implicated in aging processes. In addition, the developing interventions in this process.
- Enrichment of MAMs with proteins involved in age-related neurological and metabolic disorders highlights their significance in disease pathogenesis.

Nevertheless, the precise localization of certain proteins within MAMs and whether their presence in the MAMs fraction is a result of fractioning techniques remain subjects of debate. Despite these uncertainties, targeting MAMs structure, function, and dynamics could offer promising therapeutic strategies for various diseases and potentially promote longevity. Further research in this area is warranted to elucidate the role of MAMs in aging and their associated pathologies.
